# Combined anterior cruciate and lateral collateral ligaments reconstruction with ipsilateral hamstring autograft: surgical technique

**DOI:** 10.1186/s13018-022-03358-0

**Published:** 2022-10-27

**Authors:** Sérgio Rocha Piedade, Carlos Górios, Filippo Migliorini, Nicola Maffulli

**Affiliations:** 1 Exercise and Sports Medicine, Department of Orthopaedic, Rheumatology, and Traumatology, University of Campinas UNICAMP, School of Medical Sciences, Campinas, Brazil; 2 Centro Universitário São Camilo, Ipiranga, São Paulo Brazil; 3 Department of Orthopaedic, Trauma, and Reconstructive Surgery, RWTH University Hospital of Aachen, 52074 Aachen, Germany; 4 Department of Musculoskeletal Disorders, School Medicine, Surgery and Dentistry, University of Salerno, 84081 Baronissi, Italy; 5 Centre for Sports and Exercise Medicine, Barts and The London School of Medicine and Dentistry, Mile End Hospital, Queen Mary University of London, London, E1 4DG UK; 6 School of Pharmacy and Bioengineering, Keele University School of Medicine, Thornburrow Drive, Stoke On Trent, England

**Keywords:** Anterior cruciate ligament injuries, Collateral ligaments, Hamstring tendons, Reconstructive surgical procedures

## Abstract

Different surgical techniques have been proposed to reconstruct combined anterior cruciate (ACL) and lateral collateral ligaments (LCL). Although these surgical techniques are reliable and reproducible, the number of autologous grafts needed for the reconstruction could be a limiting factor, especially when patients present with multi-ligament knee injuries and the posterior cruciate ligament is also torn. In addition, some of these techniques are not easy to master and have a steep learning curve. We present a surgical procedure that has been used over the last 18 years to reconstruct combined ACL and LCL injuries and has become a reproducible, feasible and time-efficient procedure to approach combined ACL and LCL injuries using an ipsilateral hamstring autograft.

## Introduction

Anterior cruciate ligament (ACL) tears associated with injury to the lateral collateral ligament (LCL) of the knee are disabling [1, 2]. In these patients, surgical reconstruction is recommended [3, 4], and different surgical techniques have been proposed using two or more tendon grafts to reconstruct the torn ligaments [5–8]. The procedures proposed differ according to the number of femoral tunnels (one or two) used to reconstruct the LCL [7–10], showing advantages and disadvantages, with a steep learning curve [9–12]. Although these surgical techniques are reliable and reproducible, the number of autologous grafts needed for the reconstruction could be a limiting factor, especially when patients present with multi-ligament knee injuries, and the posterior cruciate ligament is also torn. Given the complexity of these injuries, allografts are increasingly used, as they reduce the time spent in harvesting and preparing the grafts, and donor site morbidity [4, 7, 8, 13]. However, access, availability and costs of allografts are limited in several settings [14]. We present a surgical procedure that we have used over the last 18 years to reconstruct combined ACL and LCL injuries, also in patients presenting with a bicruciate ligament injury associated with an LCL tear.

### Indications

In our practice, the diagnosis is by clinical assessment, and confirmed imaging by magnetic resonance imaging (MRI). Valgus stress radiographs are used to quantify the asymmetric lateral joint line widening.

### Surgical technique

**Fig. 1:**
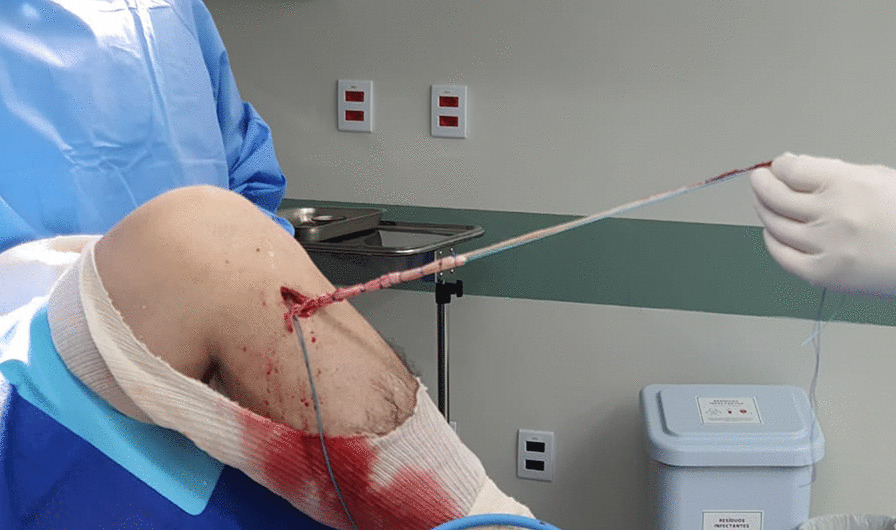
Intraoperative view of the left knee: ACL + LCL graft (double semitendinosus tendon attached to its tibial insertion sutured with a single strand of gracilis tendon)

With the patient supine and under spinal anesthesia, the knee to be operated is kept at 90° flexion with a support on the lateral aspect of the proximal 1/3 of the thigh and another one under the foot. After exsanguination, the tourniquet is inflated to 300 mmHg. A routine diagnostic arthroscopy of the knee is performed to confirm the diagnosis. If necessary, meniscal injuries are addressed. The ACL stumps are resected. The ACL femoral tunnel is performed by an outside-in ACL guide positioned at a slightly posterior point (5 mm) to the origin of the LCL in the femur, under arthroscopic control. Initially, the bone tunnel has a 6 mm diameter and will be adjusted later according to the diameter of the harvested graft. The fibular head is approached through a 6–7 cm longitudinal incision. The common peroneal nerve is identified and protected before proceeding with the dissection to bone. The diameter of the tunnel is normally 5–6 mm depending on the diameter of the gracilis graft. Through a 5 cm longitudinal incision on the pes anserinus, the gracilis and semitendinosus tendons are harvested with an open stripper, keeping them attached to their insertion on the tibia. Using the incision used to harvest the hamstring tendons, a tibial tunnel 6 mm in diameter is drilled at a 55° angle using a tibial guide under direct arthroscopic control. Again, the tunnel diameter will be adjusted after measuring the diameter of the final graft. After all bone tunnels have been drilled, the length of ACL and LCL graft are measured. Then, the gracilis tendon is detached from its tibial insertion to build the ACL and LCL grafts. According to the length of the tendons, the ACL graft could have a double or triple semitendinosus tendon plus a strand of gracilis. In contrast, the LCL has a strand of gracilis long enough to loop the fibular head and return to the femoral tunnel. The tibial and femoral tunnel diameter is adjusted according to the diameter of the final graft (Fig. 1).

**Fig. 2:**
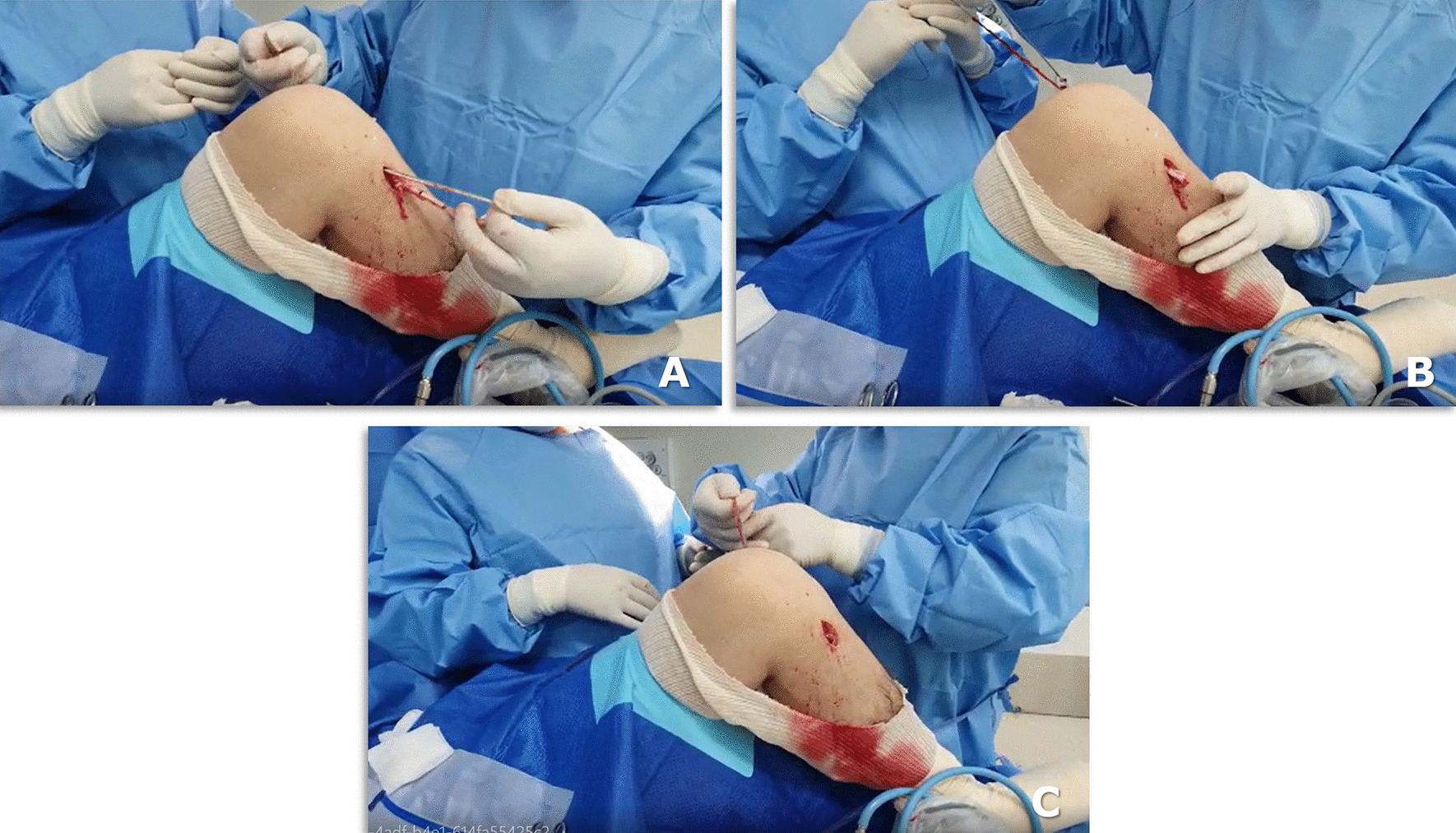
Intraoperative view of the left knee: the passage of ACL + LCL graft from the tibial to femoral tunnels

The graft is shuttled into the knee joint through the tunnels using an Ethibond 5.0 suture (Fig. 2a–c), and the knee is flexed and extended 20 times to tension the graft.

**Fig. 3:**
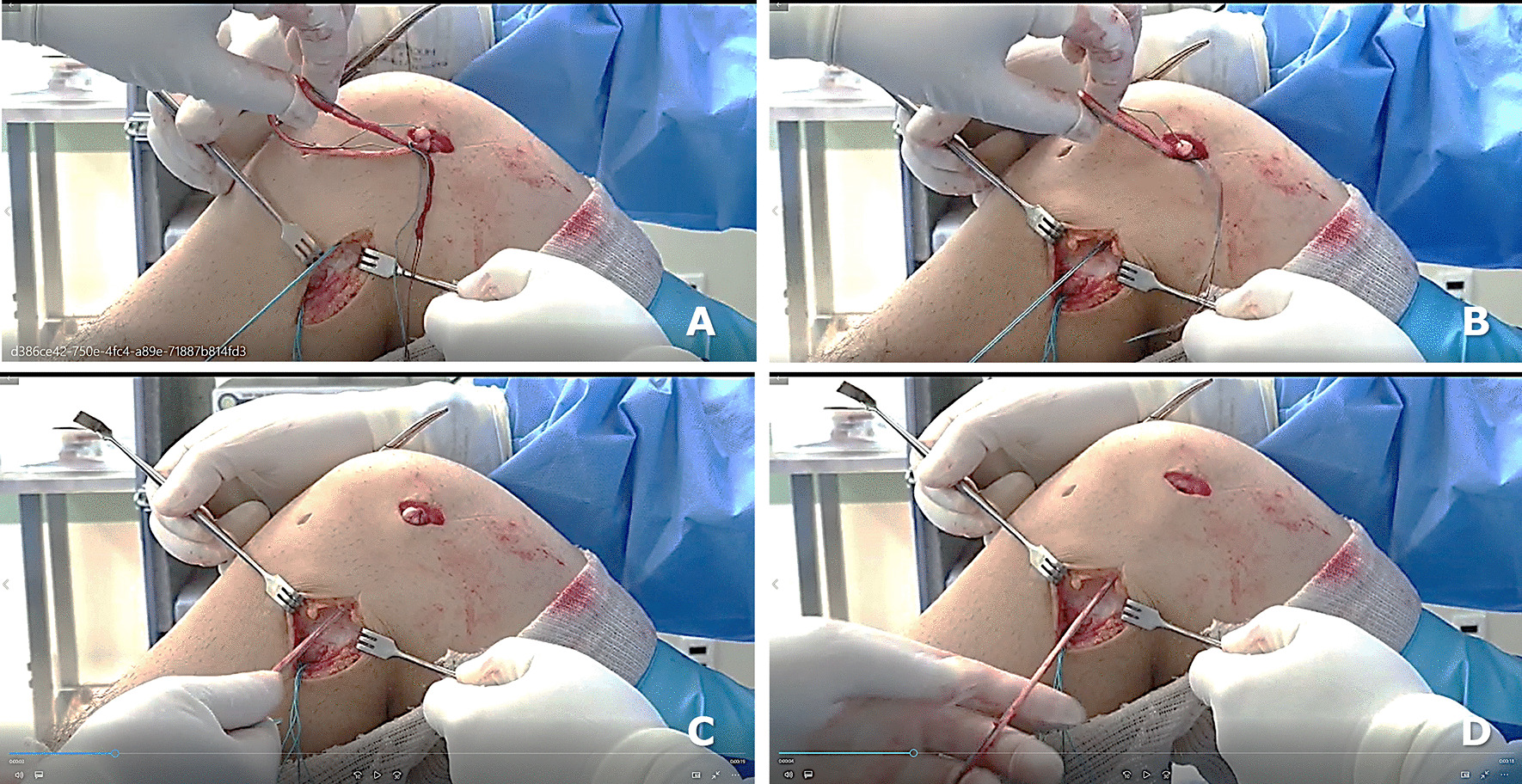
Intraoperative view of the lateral side of the left knee: the free end of graft (single gracilis strand) emerges from the ACL femoral tunnel (**a**), passing under the fascia and parallel to the fibula (**b**), emerging in the level the fibular head after graft tensioning (**c, d**)

The graft fixation starts on tibial side, followed by fixation on the femoral side using an interference screw 1 mm in diameter greater than the respective tunnel. At the femoral side, the free end of gracilis strand (Fig. 3a) is passed subcutaneously under the fascia lata fascia and parallel to the fibula to emerge in the level the fibular head (Fig. 3b–d).

**Fig. 4:**
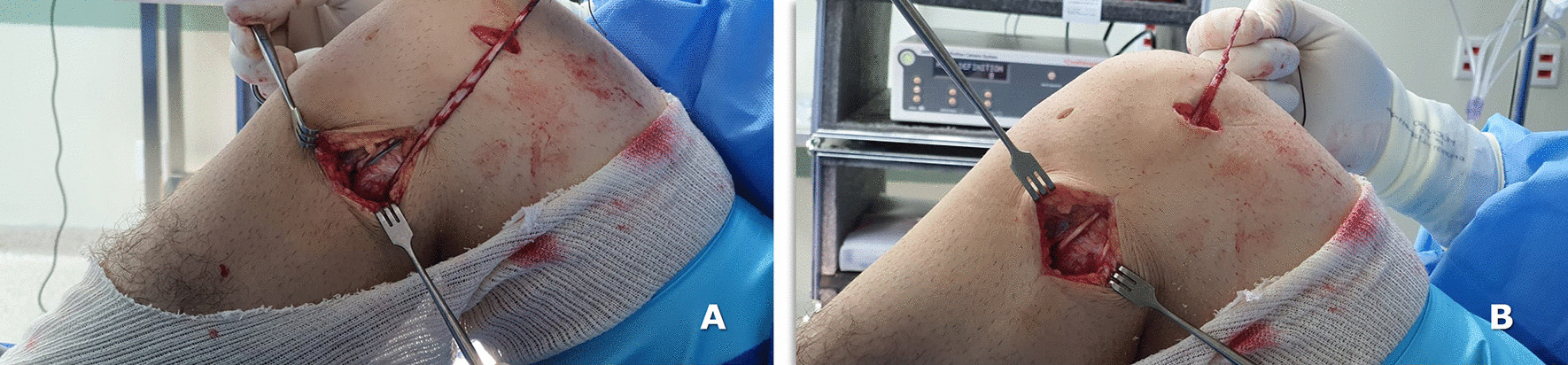
Intraoperative view of the left knee: the loop-free end of ACL + LCL graft passing into the fibular head tunnel and its fixation with a 6-mm interference screw

The single strand gracilis tendon is passed in the fibular head in an anterior to posterior direction, cycled and fixed in the fibular head with an interreference screw with the knee flexed at 45°. The remaining part of the gracilis tendon graft is sutured to the descending portion of the graft (Fig. 4a, b).

**Fig. 5:**
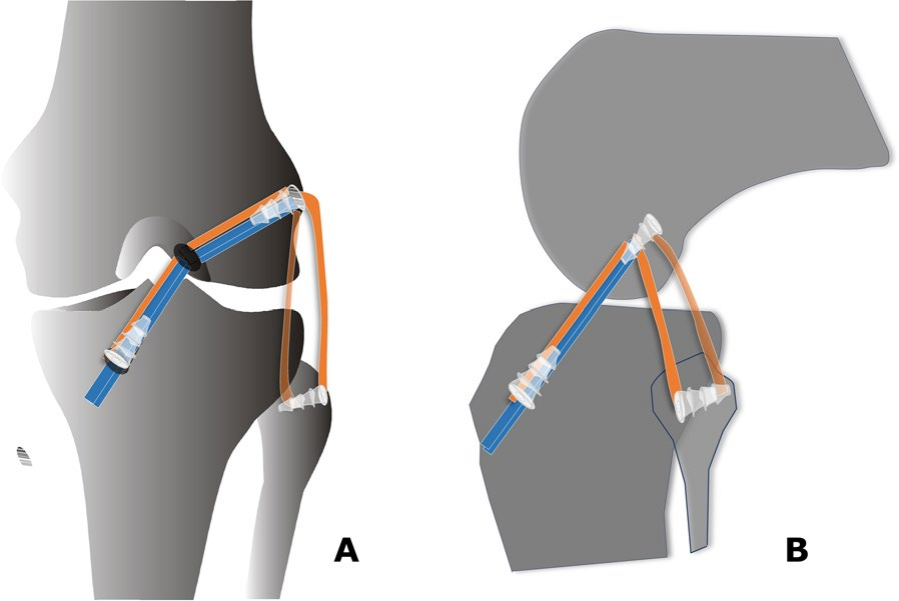
The ACL and LCL grafts fixation into the tibia, femoral and fibular head tunnels of the left knee by using absorbable interference screws, in anteroposterior (**a**) and lateral (**b**) views

The antero-posterior and lateral stability of the knee is tested and confirmed using the Lachman test and varus stress test, respectively. The tourniquet is released, accurate hemostasis is performed, and the wound is sutured in a standard fashion. The knee is bandaged and immobilized in a full extension brace. The surgical procedure is schematically showed in Fig. 5.

## Postoperative management

In the first two weeks, the knee is kept in full extension, and full weight-bearing is allowed keeping the knee immobilized in the brace. Using crutches is not mandatory, but patients feel more confident using them for the first 2 or 3 postoperative weeks. Cryotherapy and isometric exercises are introduced from the first postoperative day. The sutures are removed after two weeks.

The brace is gradually unlocked 3 or 4 weeks postoperatively, and motion is restricted to 45° in flexion until the sixth postoperative week. After 6 weeks, the brace is removed, and rehabilitation focuses on progressively gaining the full range of motion, which is expected to be achieved at around three months postoperatively.

## Key points of this surgical technique

- perform arthroscopic and clinical assessment of LCL insufficiency applying a knee varus stress with the knee extended and the figure-of-four position.
- use an open stripper to harvest the graft, keeping it attached to its tibial insertion,
- prepare both grafts using an Ethibond 5.0 suture thread to measure the length of both grafts from tunnel to tunnel (tibia—femur—fibular head—femur)
- start performing a 6 mm bone tunnel diameter, and then adjust the diameter according to the diameter of the graft

## Discussion

Combined reconstruction of ACL and LCL is technically demanding. Some authors have proposed surgical techniques involving more than one femoral tunnel to perform a combined ACL and LCL reconstruction and use different tendon grafts [5–9, 11]. These procedures are not easy to master, and especially at the beginning of one’s experience surgeons may encounter ligament bone tunnel confluence, impairing or preventing proper graft fixation, and consequently compromising outcomes. We are aware that variations to the surgical technique reported in the present manuscript are possible. For example, one of us drills the tibial and femoral tunnels only after the graft has been harvested and prepared, so that only one passage is performed, and the diameter of the tunnels does not need to be adjusted secondarily. We pass the gracilis graft in the fibular head in an anterior to posterior direction. It is obviously possible to pass in in a posterior to anterior direction, and at present we do not know whether one direction of passage results in more favorable biomechanical and clinical outcomes. Surgical techniques that simplify the procedure and use autologous grafts to reconstruct two or more ligaments are a promising alternative, especially for surgeons, medical centers and countries where allografts are unavailable or difficult to procure. In the last 18 years, the technique presented in this article has become a reproducible, feasible and time-efficient procedure to approach combined ACL and LCL injuries using an ipsilateral hamstring autograft.

## Conclusion

This surgical technique broadens the use of autografts to reconstruct combined ACL and LCL injuries and is an exciting alternative for bicruciate ligament reconstruction associated with an LCL tear.

## Data Availability

The datasets generated during and/or analyzed during the current study are available throughout the manuscript.

## Abbreviations

ACL: Anterior cruciate ligament
LCL: Lateral collateral ligament
